# Effect of *Nigella sativa* on Selected Gastrointestinal Diseases

**DOI:** 10.3390/cimb45040198

**Published:** 2023-04-03

**Authors:** Sara Jarmakiewicz-Czaja, Magdalena Zielińska, Kacper Helma, Aneta Sokal, Rafał Filip

**Affiliations:** 1 Institute of Health Sciences, Medical College of Rzeszow University, 35-959 Rzeszow, Poland; 2 Institute of Medicine, Medical College of Rzeszow University, 35-959 Rzeszow, Poland; 3 Department of Gastroenterology with IBD Unit, Clinical Hospital No. 2, 35-301 Rzeszow, Poland

**Keywords:** anti-inflammation, bioactives, intestinals, liver, nigella sativa, pancreas, stomach

## Abstract

*Nigella sativa L.* (family *Ranunculaceae*), also known as black cumin, has been used in cuisine around the world for many years. Due to its health-promoting properties, it can be used not only in the food industry but also in medicine. The main bioactive compound contained in the black cumin extract is thymoquinone (TQ), which has a special therapeutic role. The results of research in recent years confirmed its hypoglycemic, hypolipemic, and hepatoprotective effects, among others. In addition, the results of laboratory tests also indicate its immunomodulatory and anticancer effects, although there is still a lack of data on the mechanisms of how they are involved in the fight against cancer. Including this plant material in one’s diet can be both an element of prophylaxis and therapy supporting the treatment process, including pharmacological treatment. However, attention should be paid to its potential interactions with drugs used in the treatment of chronic diseases.

## 1. Introduction

**Figure 1:**
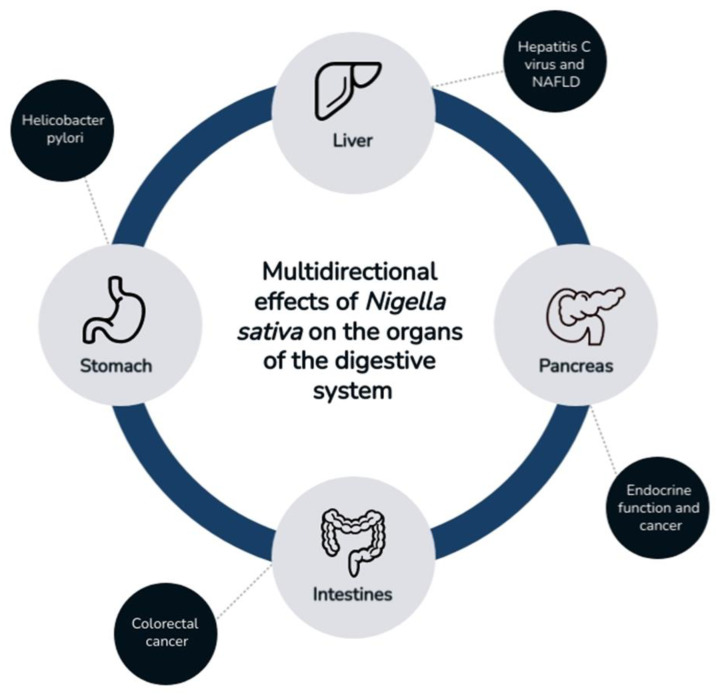
Effect of Nigella sativa on the digestive tract.

*Nigella sativa L.* (family *Ranunculaceae*), also known as black cumin, is an annual plant. It grows mainly in selected parts of Asia and southern Europe. The beneficial properties of black cumin were already known in ancient times [1]. It consists of non-volatile compounds, such as tannins or flavonoids, and volatile compounds, such as terpene compounds [2]. It shows a wide spectrum of activity in the food industry as a spice, as well as in the medical industry as a result of its antioxidant properties. The active compounds contained in *Nigella sativa* (NS) show strong pharmacological potential; this is why they can potentially be used as valuable components of drug-supporting therapies for various diseases [3]. In several scientific reports, researchers described the beneficial effects of black cumin on many diseases. By modulating the profile of inflammatory cytokines, NS shows antioxidant activity, even in chronic inflammatory conditions. It displays a wide spectrum of actions, for example, protective effects in cardiovascular diseases, i.e., high blood pressure, and showing hypolipidemic effects [4,5,6]. It has a positive effect in metabolic diseases, such as diabetes and obesity, and in various types of cancer, such as multiple myeloma and lymphoma [7,8,9,10,11]. In addition, immuno-, nephro-, and neuroprotective effects were reported [3,12,13]. NS also shows beneficial effects on the gastrointestinal tract, such as the stomach, pancreas, liver, and intestines. Due to the numerous medicinal properties of nigella, the main objective of this review was to present published scientific reports on the effects of NS on the gastrointestinal tract (Figure 1).

## 2. Chemical Composition of *Nigella sativa*

Several bioactive compounds found in different varieties of *Nigella sativa* are described in the literature. There may be slight differences in its phytochemical composition, depending on the growing region, maturity stage, processing, or storage methods. The main active compound contained in black cumin extract is thymoquinone (TQ), which has a particular therapeutic role [14]. Other important constituents include thymohydroquinone (THQ), dithymoquinone (DTQ), thymol (THY), p-cymene, carvacrol, t-anethole, α-pinene, and several other compounds that constitute terpenes and terpenoids, which are the main chemical group of Nigella [15,16]. Trace ingredients, i.e., carvone, limonene, and citronellol, were also found [17]. Other key phytonutrients of nigella are sterols (β-sitosterol, stigmasterol, campesterol, 5-avenasterol) and saponins (e.g., triterpenes-alpha-hederin), phenolic compounds, and alkaloids. Nigella seeds contain isoquinoline alkaloids (nigellicymine and nigellicymine N-oxide), diterpene alkaloids (nigellamine), and indazole alkaloids (nigellidine and nigellicin) [5]. Depending on the variety, *Nigella sativa* contains (on a dry matter basis) water (3.8–7.0%), proteins (18.59–31.2%), fats (22.0–56.4%), carbohydrates (24.9–40%), dietary fiber (3.7–4.7%), both soluble (20.5–27.1 g/100 g) and insoluble fractions (6.5–8.9 g/100 g), fat-soluble vitamins (mainly α-, β- and γ-tocopherols), water-soluble vitamins (B1, B3, B6, B9), and minerals (potassium, iron, calcium, zinc, phosphorus, magnesium, copper) [18]. Current findings show that glutamic acid is the predominant amino acid of NS, followed by aspartic acid, arginine, leucine, and glycine. Nigella seeds contain relatively high amounts of alanine, isoleucine, serine, threonine, and phenylalanine, while the cysteine content is low [19]. The major unsaturated fatty acids are linoleic acid and oleic acid, and the predominant saturated fatty acid is palmitic acid [20,21]. Myristic, palmitoleic, stearic, linolenic, arachidic, and lignoceric acids were also detected [22]. The content of tocopherol isomers in black cumin may be influenced by the extraction methods used. Their total content in black cumin oil ranges from 9.15 to 27.92 mg/100 g [23]. According to Albakra et al., the levels of α-tocopherol, β-tocopherol, and γ-tocopherol in nigella seed oil were 25.59 mg/100 g, 14.21 mg/100 g, and 242.83 mg/100 g, respectively [9]. In addition, a total of 19 polyphenols were identified in the seeds. These include caftaric acid, gentisic acid, caffeic acid, chlorogenic acid, p-coumaric acid, ferulic acid, quercetin, kempferol, and apigenin. Of these, quercetin and kempferol were found in the highest amounts [24]. *Nigella sativa,* due to its variety of organic compounds and their properties, shows potential in the prevention and support of the treatment of many gastrointestinal disorders.

## 3. Effects of *Nigella sativa* on the Pancreas

The pancreas is one of the organs that influence the functioning of the entire body. It has two main functions: endocrine (production of hormones, such as insulin and glucagon) and exocrine (functions as a digestive gland) [25]. The multidirectional effects of *Nigella sativa* are mainly concerned with the endocrine function of the pancreas. To date, scientific reports that focussed mainly on *Nigella sativa* seeds and their main compound, thymoquinone, indicate their antidiabetic effects and the prevention of diabetic complications [26,27,28,29].

In current reviews based on animal model studies, nigella seed extracts were shown to support the treatment of diabetes by significantly decreasing fasting blood glucose levels and glucose levels 2 h after meals, thereby decreasing glycated hemoglobin and improving insulin tolerance [12,13]. In an animal model study, Faisal Lutfi et al. evaluated the 4-week effects of TQ on glycaemic control and oxidative stress, among others [30]. The potential therapeutic effects of TQ were shown to lower fasting blood glucose levels and attenuate oxidative stress. These findings support the antidiabetic potential of TQ, where it was also shown to be effective at regenerating pancreatic β-cells and attenuating pancreatic inflammation and oxidative stress, as well as inhibiting β-cell apoptosis [31,32]. The study by Dalli et al. demonstrated the inhibitory effect of different fractions of nigella on pancreatic α-amylase and intestinal glucose absorption and concluded that *Nigella Sativa* compounds could be used as antidiabetic agents due to their nontoxic effects and high efficacy [33]. This effect was attributed to various bioactive compounds of nigella, such as thymoquinone, p-coumaric acid, naringenin, quercetin, kemferol, and linoleic acid. Similar results were obtained in an in vitro study by Varghese et al., in which a microencapsulated hydroacetone extract of *Nigella sativa* seeds was shown to have the ability to inhibit α-amylase [34]. The increase in inhibition was attributed to thymoquinone, which is degraded by light, and microencapsulation protects it from degradation and, consequently, increases α-amylase inhibition. The results of the in vitro study by Tiji et al. confirmed the benefits of using nigella seeds to support the treatment of diabetes. In addition, the inhibitory effect of *Nigella sativa* extracts and fractions on intestinal α-glucosidase and pancreatic α-amylase activity was demonstrated [35]. Furthermore, the toxicity test showed that the tested extracts were nontoxic up to a concentration of 10 g/kg, allowing for further evaluation for future potential in vivo applications. Hannan et al. also described the beneficial effects of nigella in diabetes, documenting that the antihyperglycemic effect of *Nigella sativa* seed extract in normoglycemic and diabetic animals is related to decreased intestinal glucose absorption and increased tissue glucose use, which is mediated by improved insulin release in response to blood glucose levels [36]. Therefore, hypotheses are now being formulated that TQ can be used as a natural support to treat hyperglycemia-induced insulin resistance in type 2 diabetes [37]. In a study on rats whose high-fat diet was enriched with *Nigella sativa* seed powder, Aboul-Mahasen et al. showed that there was a reduction in α-amylase and serum glucose levels and an increase in insulin levels. Furthermore, *Nigella sativa* seeds were highlighted to have antihyperlipidemic and hypoglycemic effects and can protect the pancreas from damage caused by hyperlipidemia (regeneration of the exocrine and endocrine parts of the pancreatic tissues of hyperlipidemic rats fed extra virgin olive oil or *Nigella sativa* seeds was demonstrated) [38]. Current analyses of *Nigella sativa’s* lowering of blood glucose levels in potentiating the hypoglycemic effect of drugs used to treat type 2 diabetes (pioglitazone) and the potential value of nanoemulsion as a nanocarrier to increase bioavailability seem promising [39].

**Figure 2:**
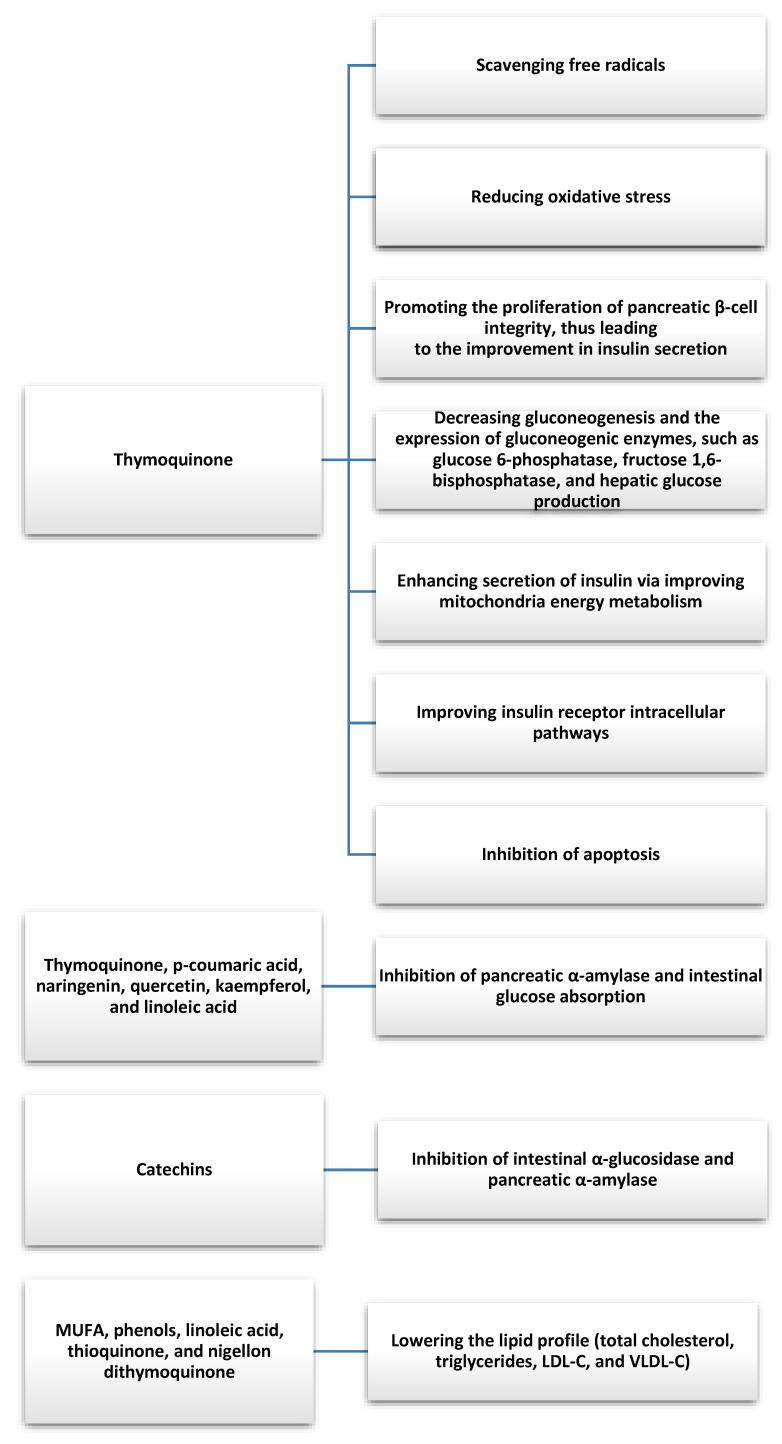
Antidiabetic and antihyperlipidemic effects of bioactive compounds and fatty acids found in *Nigella Sativa* [26,27,33,34,35,41].

There were several clinical studies that evaluated the antidiabetic effects of *Nigella sativa*. Badara et al. conducted a non-random clinical trial among patients with type 2 diabetes in which 57 patients received 2 g of *Nigella sativa* daily for one year and 57 received a placebo in conjunction with oral hypoglycemic agents [40]. Supplementation with *Nigella sativa* improved total cholesterol and its HDL fraction (significantly increased in the study group), blood pressure, and heart rate in patients with type 2 diabetes taking oral hypoglycemic agents. Kooshki et al. obtained similar results during a study in which the intervention group took 1000 mg of *Nigella sativa* oil in two capsules per day for 8 weeks [41]. The use of the nigella supplement was significantly associated with a decrease in triglycerides, total cholesterol, LDL fraction, serum C-reactive protein (CRP), and malondialdehyde, and an increase in HDL fraction cholesterol in the intervention group compared with the placebo group. A prospective and randomized clinical trial involving patients with newly diagnosed type 2 diabetes showed that the administration of metformin or 1350 mg/day *Nigella sativa* oil capsules for 3 months resulted in a reduction in fasting blood glucose levels 2 h after a meal. Glucose and glycated hemoglobin levels in the *Nigella sativa* group were found to be worse than in the metformin treatment group, but the *Nigella sativa* treatment resulted in a significant decrease in body weight, waist circumference, body mass index, fasting insulin, insulin resistance, total cholesterol, LDL fraction cholesterol, and triglycerides, which were comparable in the metformin-treated patients [42]. Similar results were obtained in a controlled study by Jangjo-Borazjani et al. involving 40 patients with type 2 diabetes and also showed that the combination of resistance training and *Nigella sativa* supplementation had a significant effect in decreasing HOMA-IR (homeostatic model assessment-insulin resistance), insulin, passive reaction (ESR), and CRP, as well as increasing HDL fraction cholesterol during the 8-week intervention [43]. Furthermore, a randomized clinical trial involving 117 obese patients with prediabetes showed that the administration of capsules containing 450 mg of *Nigella sativa* oil twice a day or 500 mg of metformin twice a day led to similar improvements in anthropometric indices, glycemia, lipid profile, and inflammatory parameters (TNF-α (tumor necrosis factor-α) levels decreased significantly) [44]. The results of the study indicate that *Nigella sativa* may be an important factor in complementary therapy with other drugs for the treatment of diabetes and the prevention of diabetic complications. In addition, the results show a protective effect on the cardiovascular system in patients with type 2 diabetes by improving, among other things, the lipid profile, glycemia, and anti-inflammatory effects (Figure 2).

For diseases related to the exocrine function of the pancreas, thymoquinone is mainly effective at reducing pancreatic α-amylase levels, regenerating pancreatic β-cells, and alleviating pancreatitis and oxidative stress [25,38]. In addition, it is beneficial in reducing the factors associated with chronic pancreatitis (which may be one of the causes of pancreatic cancer) mentioned earlier, such as reducing triglycerides, weight loss, and waist circumference [29].

### Pancreatic Cancer

**Figure 3:**
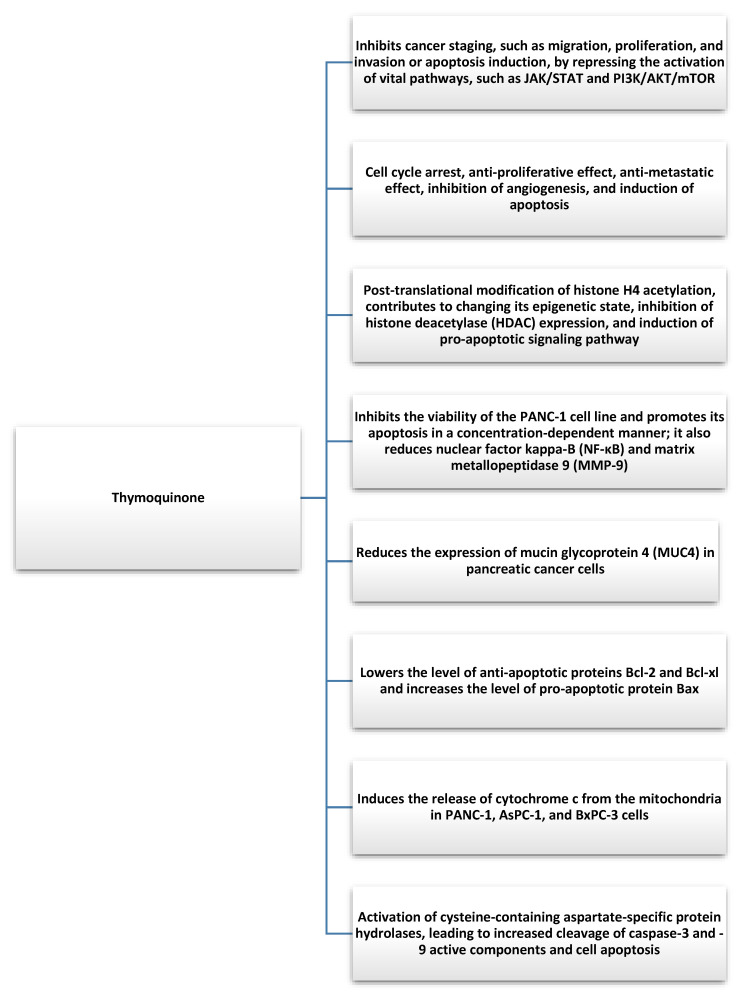
Anticancer effects of thymoquinone found in *Nigella Sativa* [49,50,52,53,55].

Pancreatic cancer is one of the most common cancers worldwide and is characterized by a particularly poor prognosis, high metastatic rates, mortality, and resistance to chemotherapeutic drugs [45]. It develops as a result of excessive proliferation and DNA mutations in pancreatic cells. These mutations cause uncontrolled growth of pancreatic cells, leading to the appearance of cancerous tumors and causing the actual cells to lose their function [46]. The causes of pancreatic cancer are not fully understood, but the following risk factors were identified: age (usually 50–70 years), smoking, family history of pancreatic cancer, diabetes, chronic pancreatitis, lifestyle, and obesity [47]. TQ is not harmful to human cells and has shown beneficial antitumor effects in various cancers, including pancreatic cancer [48]. Mechanisms underlying the TQ-mediated apoptosis of pancreatic cancer cells based on in vivo and in vitro experiments include cell cycle arrest, the antiproliferative effect, the antimetastasis effect, the inhibition of angiogenesis, and the induction of apoptosis [32]. Furthermore, TQ mediates the post-translational modification of histone H4 acetylation, contributes to the alteration of its epigenetic state, inhibits histone deacetylase (HDAC) expression, and induces a pro-apoptotic signaling pathway [49,50]. More than 15 years ago, scientists confirmed that TQ can limit the proliferation of the pancreatic cancer cell line PANC-1 [51]. Furthermore, TQ can inhibit the viability of the PANC-1 cell line and promote its apoptosis in a concentration-dependent manner, as well as downregulate nuclear factor kappa-B (NF-κB) and matrix metallopeptidase 9 (MMP-9) [52,53]. In addition, TQ can reduce mucin glycoprotein 4 (MUC4) expression in pancreatic cancer cells, contributing to the regulation of differentiation, proliferation, reduced migration of pancreatic cancer cells, and chemoresistance [54]. Furthermore, Mu et al. analyzed the antitumor effects of thymoquinone and gemcitabine and found that TQ downregulates the antiapoptotic proteins Bcl-2 and Bcl-xl, increases the pro-apoptotic protein Bax, induces the release of cytochrome c from the mitochondria of cells with PANC-1 AsPC-1 and BxPC-3, and activates a family of aspartate-specific protein hydrolases containing cysteine in a dose-dependent manner, resulting in increased cleavage of active components of caspase-3 and -9 and cell apoptosis [55] (Figure 3). In this context, particular attention should be paid to the occurrence of drug resistance in the treatment of pancreatic cancer, such as gemcitabine (GEM). The most common type of pancreatic cancer is pancreatic ductal adenocarcinoma (PDAC), for which gemcitabine and gemcitabine-based chemotherapy are insufficient [56]. It was shown that pretreatment of pancreatic cancer cells with TQ can increase the sensitivity of cells to the drug [57]. Based on the results of the study, it was hypothesized that the combination of gemcitabine with TQ could improve the benefits of the drug treatment [58]. Karki et al. showed that TQ can also exert a synergistic effect with juglone, which is another cytotoxic molecule for pancreatic cancer cells [59]. Furthermore, TQ in pancreatic cancer also exhibits powerful anti-inflammatory effects, reducing the synthesis of inflammatory cytokines, such as monocyte chemotactic protein 1 (MCP-1), TNF-α, interleukin, and cyclooxygenase-2 (COX-2) in pancreatic ductal cell carcinoma, depending on the dose and time [32]. Preclinical in vitro and in vivo studies demonstrated the therapeutic ability of TQ against pancreatic cancer, but data are still limited. TQ was confirmed as a promising anticancer agent with different molecular mechanisms of action underlying its multidirectional tumor inhibition. The synergistic effect of TQ on drugs may also contribute to the resolution of drug resistance in pancreatic cancer. Currently, research is limited mainly to laboratory studies; however, the discovery of TQ carriers (nanoparticle preparations of TQ) opens the way to their clinical applications [60,61]. More research is needed to effectively plan the intervention, assess the safety of TQ, and identify the mechanisms involved in pancreatic cancer.

## 4. Effects of *Nigella sativa* on the Liver

In a study by Kanter et al., the authors analyzed the effects of NS on the action of selected liver enzymes in animal models with the introduction of carbon tetrachloride (CCl4) treatment. Treatment of rats with CCl4 for 45 days decreased the antioxidant enzyme levels and increased the levels of selected liver enzymes (ALP, alkaline phosphatase; AST, aspartate aminotransferase; ALT, alanine aminotransferase) and lipid peroxidation. On the other hand, from days 46 to 90, treatment with *Nigella sativa* L. or *Urtica dioica* L. (as a single substance or in combination) was included, which reduced the liver enzyme concentrations and the degree of lipid peroxidation and increased the levels of antioxidant enzymes. In addition, the inclusion of NS in therapy may exhibit a treatment-induced weight loss effect [62]. In another study, the authors evaluated the effects of NS on metabolic disorders that were induced by bisphenol A (BPA) in rats. When NS was administered with BPA or thymoquinone with BPA, a decrease in selected liver enzymes was observed [63].

In human studies, Hussaina et al. also observed a reduction in liver enzymes (AST, ALT) after supplementation with 1 g of NS twice a day in patients with non-alcoholic fatty liver disease (NAFLD). Furthermore, the body weight of patients with a high BMI (body mass index) was reduced, and in more than half of the patients, the parameters indicative of liver steatosis improved. The researchers pointed out that it is worthwhile to include treatment as early as the early stages of NAFLD [64]. In another study involving 40 men diagnosed with NAFLD who were divided into two groups (NS and placebo), liver parameters, such as AST and ALT, were reduced in the NS group after supplementation with 1 g of NS per day for 8 weeks compared with the placebo group. However, for GGT (gamma-glutamyl transpeptidase), the researchers did not observe a significant difference. In addition to improving the levels of selected liver enzymes, the authors pointed to significant reductions in inflammatory factors, i.e., CRP (C-reactive protein), IL-6 (interleukin-6), and TNF-α [65]. Darand et al. recommended supplementation of up to 2 grammes of NS per day for patients with NAFLD to reduce inflammation [66]. In a meta-analysis, Razmpoosh et al. found that there were no interventive dosing effects of NS on AST and ALP levels, while an association with ALT values was demonstrated [67]. Khonche et al. studied the efficacy and safety of black cumin oil in NAFLD. The study enrolled 125 patients, who were assigned to two groups (with NS supplementation and a placebo group). They took 5 mL of either NS oil or placebo every 12 h for 3 months. In the NS supplementation group, researchers found a significant decrease in TG (triglyceride), LDL-C (low-density lipoprotein cholesterol), and the degree of liver steatosis compared with the placebo group [68]. In their review, Mohtashamian et al. also pointed to the hepatoprotective effects of NS by, among other things, reducing oxidative stress in hepatocytes and decreasing lipid accumulation in the liver, but they also pointed to the need for more studies to determine the effects of NS on NAFLD [69]. Other authors also pointed to significant reductions in liver steatosis through NS supplementation [70].

*Nigella sativa* not only has a protective effect on hepatic steatosis but may also show beneficial effects in hepatitis. Barakat et al. evaluated the efficacy and safety of NS in patients diagnosed with hepatitis C who cannot be treated with interferon α (IFN-α). They administered NS seed oil in capsule form (450 mg) to 30 patients three times a day after meals for 3 months. They observed a reduction in the viral load of hepatitis C (HCV) and an improvement in factors such as RBC (red blood cells), platelets, total protein, and albumin. Furthermore, postdosing NS also had an effect on lowering the glucose levels in HCV patients. The authors pointed to the role of NS in improving clinical indicators in this group of patients; therefore, the introduction of such an option into primary therapy could be considered [71]. In another study, Abdel-Moneim et al. tested the effects of NS extract and Zingiber officinale extract on HCV. Sixty patients with a 15-patient control group were included in the study. A group was given a capsule of NS extract (500 mg) twice a day for one month. They showed that NS significantly reduced viral titers and improved the overall clinical status of patients with HCV [72]. In another study, NS was administered along with antiviral drugs used in HCV patients at a dose of 250 mg per day for 8 weeks. The researchers observed an improvement in biochemical blood results and a reduction in liver enzymes. NS, through its antioxidant activity, can also reduce the adverse effects of drugs. However, further studies are needed to evaluate the impact of NS on HCV treatment [73]. Thymochonon (TQ), which is one of the components of NS, reduced liver damage by inhibiting IFN-γ (cytokine interferon-gamma) and TNF-α and blocking nuclear factor kappa-light-chain-enhancer of activated B cells (NF-κB) signaling [74]. The reduction of thymochonon inflammatory responses can occur through the degradation of IRAK1 (interleukin-1 receptor-associated kinase 1) and, consequently, lead to the reduced activity of AP-1 (activator protein 1) and NF-κB [75]. The substance also exhibits antigenotoxic effects by inhibiting the activity of cytochrome P450 1A2/A2, (CYP1A1/A2), which are involved in the biotransformation of xenobiotics [76]. NS can also be used as an adjunct to antiparasitic therapy, which could reduce liver damage caused by parasites [20]. In addition, it exhibits antifungal activity, which also has a strong effect on liver function [77]. Thymoquinone was indicated by many researchers as a chemopreventive agent and beneficial as an adjuvant to liver cancer therapy [78,79]. Therefore, new agents are being developed with the addition of NS oil to enhance anti-HepG2 cell lines [80]. The mechanism of action of NS is poorly understood, and it was suggested that thymoquinone may post-mediate an increase in miR-1-3p expression, which, by affecting levels of matrix metalloproteinase-2 (MMP2) or TIMP Metallopeptidase Inhibitor 3 (TIMP3), among others, may inhibit angiogenesis [81].

**Figure 4:**
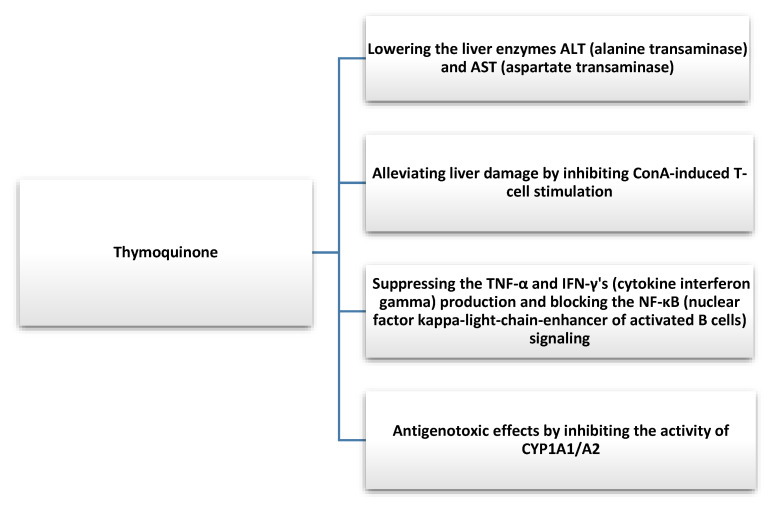
Hepatoprotective effect of thymoquinone found in *Nigella Sativa* [74,76].

NS shows a broad spectrum of action (Figure 4) on the functioning and various pathological conditions of the liver; therefore, its inclusion in basic therapy can be considered.

## 5. Effects of *Nigella sativa* on the Stomach

In animal models, both TQ and NS showed antioxidant properties that helped to lower elevated free radicals that promote the development of gastric disorders [82,83]. Using a rat model, Manjegowda et al. showed that RG-I type pectic polysaccharide of NS can stimulate a molecular signaling cascade that can enhance the ulcer healing process [84]. TQ was also found to increase NO production and gastric mucin secretion. TQ exhibits inhibitory effects on factors that may be involved in the depletion of the gastric mucosal layer, reduces gastric acid secretion by inhibiting the action of the proton pump, and decreases neutrophil invasion. It suggests that TQ may find use in conditions related to gastric hyperacidity, such as gastritis, dyspepsia, reflux, or peptic ulcers [82,85].

*Helicobacter pylori (H. pylori*) infection is one of the most common chronic bacterial infections associated with the development of gastritis and gastric ulcers [86]. In addition, it is one of the biggest risk factors for the development of gastric cancer and it was classified as a group I carcinogen to humans, which means there is convincing evidence of carcinogenicity in exposed humans. Additionally, there is evidence that *H. pylori* eradication in asymptomatic individuals reduces the risk of gastric cancer [86,87]. The antibacterial properties of NS, TQ, and thymohydroquinone were demonstrated in in vitro studies against both Gram-negative and Gram-positive bacteria [88,89]. NS also shows antimicrobial activity against some multidrug-resistant pathogenic bacteria [90].

While many of the studies on NS used animal models, a few human studies that evaluated the effect of NS on *H. pylori* eradication were also published. Salem et al. conducted a study involving 88 adult patients (56 females and 32 males) with non-ulcer dyspepsia and confirmed *H. pylori* infection [91]. Participants were divided into four groups: (1) standard triple therapy (TT)—clarithromycin, amoxicillin, and omeprazole; (2) 1 g NS powder + 40 mg omeprazole (OM); (3) 2 g NS powder + OM; and (4) 3 g NS powder + OM. TT was applied for one week and the other therapies for four weeks. The TT group, which received one of the standard therapies against *H. pylori*, achieved an 82.6% eradication rate. The 2 g NS + OM group achieved a statistically comparable result to the TT group (66.7%). The other groups were significantly less effective at eradicating *H. pylori* than TT (1 g NS—47.6%, 3 g NS—47.8%) [91]. It is worth noting that eradication was more effective in the group with a lower supply of NS (2 g vs. 3 g). Improvement of dyspeptic symptoms was similar in all groups, which was probably related to proton-pump inhibitor (PPI) administration. The study suggests that the administration of NS along with PPIs may have the potential to be used as *H. pylori* eradication therapy with comparable efficacy to therapy using clarithromycin and amoxicillin. Dosin is a traditional Islamic mixture of NS with honey (HNS) [92]. There are references to the use of HNS to relieve symptoms of the upper gastrointestinal tract in the literature of traditional Persian medicine [93]. In a pilot study by Hashem-Dabaghian et al., 19 patients (13 females and 6 males) with gastrointestinal complaints and *H. pylori* infection were advised to receive 6 g of HNS three times a day (6 g/day of NS seeds and 12 g/day of honey) after meals for two weeks. After the intervention, the eradication of *H. pylori* infection was observed in 57.1% (n = 8) of participants and dyspepsia symptoms reduced significantly [92]. Hanafy and Hatem demonstrated in an in vitro study that NS can exhibit a synergistic antibacterial effect in combination with streptomycin and gentamicin, as well as an additive effect with several other antibiotics, suggesting that NS administered with antibiotics can increase their efficacy [88]. Alizadeh-Naini et al. conducted an RCT that included 46 adult *H.*-*pylori*-infected patients diagnosed with functional dyspepsia. Both groups received quadruple therapy (500 mg metronizadole + 1000 mg amoxicillin + 240 mg bismuth subcitrate + 40 mg OM twice daily) for two weeks. The treatment group additionally received 2 g NS daily for 8 weeks, and the placebo group received 2 g placebo daily for 8 weeks. The *H. pylori* eradication rate was significantly increased in the NS group compared with the placebo group (*p* = 0.01). After the intervention, 45.45% (n = 10) of participants were still *H.*-*pylori*-positive in the placebo group compared with 12.5% (n = 3) in the NS group [94].

In 2015, Mohtashami et al. conducted a study evaluating the effectiveness of HNS in patients with functional indigestion [93]. The RCT involved 70 participants (35 each in the experimental and control groups). Patients were assessed for dyspepsia severity using the Hong Kong index of dyspepsia questionnaire (HKDI). After 8 weeks of intervention, the mean HKDI score was significantly lower in the group that received 5 mL of NS oil with honey and water compared with the placebo group, which received only honey with water (*p* < 0.001). Furthermore, the presence of *H. pylori* infection was verified before and after the test using a urease test. The *H. pylori* eradication rate was significantly increased in the NS group compared with the placebo group. No significant adverse effects were noted during the course of the study, and its results provide a basis for further research with a larger sample size [93].

**Figure 5:**
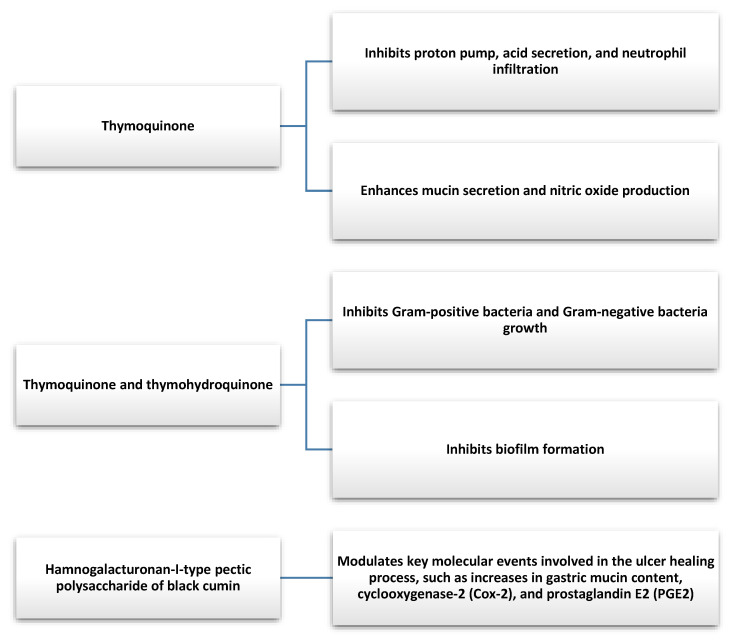
Gastroprotective effect of bioactive compounds found in *Nigella Sativa* [89,90].

NS seems to have potential clinical applications in gastric diseases (Figure 5), particularly in the treatment of diseases associated with *H. pylori* infection. Further well-designed clinical trials verifying the efficacy of using NS in combination with antibiotics for *H. pylori* eradication may be warranted, especially given the increasing antibiotic resistance and declining eradication rates of currently used therapies [86]. It should be noted that there is currently insufficient evidence to establish a recommended NS dose. Various doses and forms of administration of this agent were used in clinical trials, often in combination with another biologically active substance.

## 6. Effects of *Nigella sativa* on the Intestines

The effects of nigella were also confirmed in studies on its effects on the gut. Shahid et al. investigated the effectiveness of NS oil and its main bioactive component, namely, thymoquinone, on the intestinal tract in animal models. They administered cisplatin(CP) to rats, which induced the overproduction of ROS (reactive oxygen species), which is associated with increased oxidative stress. After the NS supply, the researchers noticed a reduction in brush-border membrane enzymatic activity in isolated brush-border membrane vesicles. In addition, both enzymatic and non-enzymatic parameterizations of the antioxidant defense of the intestinal mucosa improved [95]. Such effects of NS were confirmed by histological and biochemical studies, indicating the potential of NS for clinical use [96]. The mechanism of the anti-inflammatory effect of TQ was presented by Venkata-raman et al. They showed that TQ exhibits PPAR-γ (peroxisome proliferator-activated receptor gamma) agonist through the induction of PPAR-γ promoter activity. Furthermore, they pointed to the suppression of MAPK kinases (mitogen-activated protein kinase) and a decrease in the activity levels of NF-κB signaling pathways, with a concomitant increase in IκB (inhibitor of nuclear factor kappa-B) expression. Due to its potent anti-inflammatory effects, the authors indicated that TQ may be considered a potential adjunctive therapy for inflammatory bowel disease [97]. In another study using animal models, El-Sheikh et al. attempted to investigate the mechanisms of action of TQ in counteracting toxicity with methotrexate treatment. The administration of methotrexate resulted in the formation of non-villiform villi and intestinal crypts, which may be a risk factor for the development of celiac disease. Furthermore, catalase activity and glutathione concentrations decreased, with a concomitant increase in malondialdehyde (MDA), indicating the development of significant oxidative stress. In the group of rats in which methotrexate treatment was accompanied by a TQ supply, increased intestinal GSH (reduced glutathione) and catalase concentrations were observed. Researchers also observed lower total nitrite/nitrate levels in the group treated with methotrexate supplemented with TQ compared with the group that received the drug alone [98]. TQ can also counteract the effects of gamma radiation by reducing superoxide dismutase (SOD) and MDA [99]. TQ also exhibits apoptotic effects by increasing the expression and activity of caspase-3 in proliferative MCF7 and HCT116. Thus, it may have applications in the treatment of aging breast and colon cancer cells [100]. The beneficial effects of NS on intestinal toxicity induced by cadmium loading were also documented. After exposure to cadmium, NS can restore the normal expression of MUC2 (mucus glycoprotein 2) expression in the colon, increase IL-2 (interleukin-2), and downregulate TNF-α [101]. Furthermore, due to the delay in digestion and absorption of carbohydrates in the intestinal tract, NS can exhibit antihyperglycemic effects, supporting the treatment of diabetes [33,102].

## 7. Safety

NS toxicity appears to be at a low level, but it should be highlighted that safety data is mainly based on preclinical studies using TQ. The review by Mashayekhi-Sardoo et al. summarized the in vivo toxicological profile of TQ. The degree of toxicity of TQ highly depends on the dose and route of administration, and TQ itself appears to be relatively safe when administered orally. LD_50_ for TQ ranges from 250 to 794 mg/kg in rats and from 300 mg/kg to 2400 mg/kg in mice when administered orally. LD_50_ for TQ administered i.p. was 57 mg/kg in rats and 90.3 to 104 mg/kg in mice. Oral TQ is safer than i.p., as it can undergo biotransformation to produce less toxic metabolites in the GI tract or become metabolized in the liver, whereas the i.p. administration of TQ makes its transport into the systemic circulation easier, which enhances its toxicity [103]. It is estimated that the LD_50_ values for TQ taken orally can be about 100–150 times higher than the doses needed to achieve therapeutic effects [104].

Severe side effects of NS are rarely observed in human studies [105]. In the Mohtashami et al. study, some patients experienced only mild adverse events, including nausea and bloating in both treatment (5 mL NS oil + honey + water) and placebo (honey + water) groups. Moreover, some participants experienced a burning sensation in the treatment group. The difference in the incidence of these complaints between the groups was not statistically significant [93]. In a study by Barakat et al. (n = 30), one case of epigastric pain and five cases of hypoglycemia (two of whom had diabetes) were reported during the NS oil treatment in patients with HCV [71]. A recent RCT that involved 60 healthy subjects (n = 29/placebo group) indicated that taking 200 mg/adult/day of TQ-rich NS oil formulation for 90 days was not associated with any significant side effects. No severe adverse effects nor any alterations in the hematological parameters were reported [106].

## 8. Drug Interactions

Polypharmacy (PP) may be associated with a higher risk of drug interactions, adverse reactions, hospitalizations, and mortality [107]. It occurs most often in the elderly, especially in hospitalized patients and those with comorbidities [108]. Patients with chronic diseases may be especially vulnerable to PP. In the context of GI diseases, it is worth highlighting IBD, which is often associated with comorbidities such as cardiovascular diseases, rheumatological diseases, acid-related disorders, autoimmune diseases, and psychiatric disorders. PP prevalence appears to be similar in IBD and other chronic diseases. According to Mesonero et al., it occurs in one in five patients in the general population and affects 48% of patients over 62 years of age. In other studies, PP defined as the administration of ≥5 drugs occurs in up to 49.8% of patients with Crohn’s disease and 29.8% of patients with ulcerative colitis [109]. The inclusion of over-the-counter drugs and supplements may contribute to increased PP prevalence in patients with chronic diseases; therefore, obtaining accurate information from the patient about the medications and supplements used, including NS, is important.

NS appears to be able to interact with various medications. In vitro studies demonstrated that TQ can significantly inhibit the activity of CYP1A2, CYP2C9, CYP3A4, and CYP2D6 enzymes in human liver microsomes [109]. This suggests that NS may interact with drugs that are metabolized by these enzymes. There are several reports in the literature regarding the potential interactions of NS with drugs used in IBD. CYP3A4 is the important enzyme responsible for the metabolism of most corticosteroids, such as budesonide, prednisone, and methylprednisolone. CYP3A4 inhibitors such as ketoconazole can inhibit budesonide and methylprednisolone metabolism suggesting that TQ could also have some inhibitory effect [110,111]. Cyclosporine (CsA) and tacrolimus are metabolized by CYP3A enzymes in the liver and small intestine. Both are the substrates for P-glycoprotein (P-gp). Inhibitors and inducers of CYP3A or P-gp may disrupt the metabolism of CsA and tacrolimus, which can affect their blood concentrations [112,113]. In an in vivo study, the oral administration of 200 NG extract for 8 days in rabbits appeared to significantly reduce C_max_ and AUC_0–t_ of CsA [112]. In Alrashedi et al.’s study, TQ administered to rats reduced the oral bioavailability of CsA but did not interfere with the i.p. bioavailability of CsA. Interestingly, TQ administration significantly attenuated the renal toxicity and diabetogenic effect induced by CsA [113].

## 9. Conclusions

Increasingly used as a spice in kitchens around the world, black cumin, due to its immunomodulatory properties, has potential applications in the prevention and treatment of many conditions accompanied by chronic inflammation.

There is ample evidence of its anticancer, anti-inflammatory, antiparasitic, cholagogic, hepatoprotective, and anti-ulcer effects (Table 1). Positive effects from its use were also observed regarding improving lipid profile and body composition parameters in patients with type 2 diabetes and liver steatosis, such as body weight and waist circumference, making it useful for the specific prevention of metabolic syndrome, the occurrence of which also increases the risk of developing the aforementioned diseases. Therefore, it seems reasonable to include this ingredient in the diet as an element of therapy supporting the treatment process, including drug treatment, while paying attention to its potential interactions with drugs used in the treatment of chronic diseases.

## Figures and Tables

**Table 1:** Summary of the effect of Nigella Sativa on gastrointestinal diseases.

Organs	Disease	Effects of Black Cumin	Conclusion
Stomach	Peptic ulcer disease	- Reduction in gastric acid secretion. - Decrease in neutrophil invasion. - Enhancement in ulcer healing in rats. - Potential anti-*H.*-*pylori* properties in humans.	- NS shows potential anti-ulcer activity, but further clinical trials verifying the efficacy of NS are needed to determine the appropriate dose and safety when used in combination with antibiotics.
Pancreas	Type 2 diabetes	- Nigella sativa has antihyperlipidemic and hypoglycemic effects.	- NS may be an important factor in complementary therapy with other drugs used in the treatment of diabetes and the prevention of diabetic complications. - NS shows cardiovascular protective effects in patients with type 2 diabetes by improving lipid profile, glycemia, and anti-inflammatory effects.
	Pancreatic cancer	- Several mechanisms underlying apoptosis of pancreatic cancer cells were described regarding TQ-mediated pancreatic cancer. - It was shown that pretreatment of pancreatic cancer cells with TQ can increase the cells’ sensitivity to pharmacotherapy.	- Further research is needed to effectively plan the intervention and evaluate the safety of TQ and mechanisms involved in the fight against pancreatic cancer.
Liver	Nonalcoholic fatty liver disease (NAFLD)	- Increased levels of antioxidant enzymes. - Decreased selected liver enzymes (ASP, ALT, ALP). - Reduction in lipid peroxidation - Lower body weight. - Lowered inflammatory factors (e.g., TNF-α).	- NS supplementation can improve the liver profile and reduce liver steatosis.
	Hepatitis C (HCV)	- Reduction in HCV viral load. - Improvement in some biochemical indices, e.g., RBC, albumin, and platelets. - Lowered glycemic levels in HCV patients. - Inhibition of IFN-γ and TNF-α and blocking of NF-κB signaling.	- Studies showed improvements in patients’ overall clinical condition. NS supplementation may reduce the adverse effects of drugs; therefore, this could be considered for introduction into primary therapy.
Intestines	Overall impact	- Improved both enzymatic and nonenzymatic parameters of the antioxidant defense of the intestinal mucosa. - Increased intestinal GSH and catalase levels were observed with TQ supplementation. - Showed apoptotic effects by increasing both expression and activity of caspase-3 in proliferative MCF7 and HCT116.	- May be considered as a potential adjunctive therapy for inflammatory bowel disease. - It shows an adjunctive effect in methotrexate therapy. - Participates in the therapy of aging breast and colon cancer cells.

NS—*Nigella sativa*, TQ—thymoquinone, ALP—alkaline phosphatase, AST—aspartate aminotransferase, ALT—alanine aminotransferase, RBC—red blood cells, IFN-γ—cytokine interferon-gamma cytokine, NF-κB—nuclear factor kappa-light-chain-enhancer of activated B cells, GSH—reduced glutathione, TNF-α—tumor necrosis factor-α.

## Data Availability

Not applicable.
